# Sick Chambers

**Published:** 1872-08

**Authors:** 


### HALL'S

### JOURNAL OF HEALTH.

VOL. XIX. — AUGUST, 1872. — NO. VIII.

### SICK CHAMBERS.

GOOD nursing, a judicious attention to the sick and all the surroundings of a sick bed, would cure many cases of disease, without the use of any medicine whatever; and in others, where medicine cannot be borne, in consequence of the great debility of the patient, or the exhaustion of the system, it has been successful in restoring health and life for many years. If families would heed the suggestions which follow with reasonable fidelity, the attention of a physician could often be dispensed with, to say nothing of the comfort afforded to the sick and suffering, with all their discouragements and despondencies.

1. The sick room should be up-stairs, if possible. There are certain pernicious emanations from the earth, besides the raw dampness and heaviness, which vitiate the air, and otherwise render it injurious to the invalid. If there is no second story, the higher the bed from the floor the better. Pallets on the floor, cradles, lounges, cots, and trundle-beds, should be exchanged for high bedsteads.

2. By all means place the sick in the room in the building which faces the sun for the greater part of the day, although it may not have as many windows as others, and although it may be smaller; for the sunshine keeps it light and cheerful; it keeps it warm, a healthful warmth; it keeps it dry, and besides, imparts a life to the atmosphere breathed, which of itself is a very important element in bringing back vitality to an exhausted frame. A merchant in New York city noticed, in the progress of years, that his chief clerks sickened soon after they came to him, and one or two of them died. The thought occurred to him, one day, that it might be because the apartment where the books were kept was so surrounded by buildings that the sun did not shine into any of the windows for a single moment during the day. Being a man of culture, energy, and of a generous nature, he decided instantly to have his books kept in the sunniest room in the building, with the result of the immediate and continued improvement and eventual recovery of permanent good health on the part of the person then in his employ. If, then, the want of sunshine is a cause of actual and even fatal disease to young and hearty persons, as all the young men were when they first came to the merchant, much worse, incalculably worse results would follow, if those who were already sick occupied rooms where no cheering, warming sunshine ever came.

3. The bed should be so situated as to be easily approached from both sides; and, if convenient, have the head towards the north, to enable the patient to face the sunshine, to look on the out-doors, which has of itself a cheering effect, lessens the monotony of the sick chamber, and tends to divert the mind from the condition of the body, thus allowing time to pass more pleasantly. It may be fancy, or it may be fact, that certain electrical currents are passing between the poles, and their course being longitudinal to the body, and for other considerations, may have decidedly advantageous results to the sick. Such an opinion has been given by different men, whose positions and abilities entitle them to the respect and confidence of educated people. The idea, however, may have readily arisen from the circumstance that if the sick bed has its head to the north, it gives the patient an easy opportunity of seeing the glad sunshine all the time.

4. A good ventilation, a constant supply and resupply of pure, cooling, out-door air, is of inestimable advantage to the sick. The fact may well be repeated that an English physician was called to attend a poor family of several members of different ages. One by one had been taken sick, until every member was an invalid in the depth of winter. The usual medicines seemed to fail of their ordinary good effects; one or two of the family died. Soon after that, in one of his visits, the physician's attention was drawn to the fact that a window-pane had been broken, and the family expressed great apprehension of taking cold, as their means were limited, and it had been very necessary to be economical with their fuel. To make less do, every crevice of door and window had been carefully stuffed to keep out the cold, with the result of preventing ventilation, and occasioning the rebreathing of vitiated air all the time. The physician had repeatedly drawn their attention to the importance of opening a door or window every day for a short time, even several times a day, but he had noticed that his suggestions had remained uniformly unheeded, the bugbear of expense and taking cold being ever present to the inmates. Under the circumstances he considered the breaking of the glass a piece of great good fortune, and put obstacles in the way of repair in such a way as to defer it as long as possible. The result was immediate and most striking. Within a day or two the invalids began to improve, and all of them eventually recovered.

5. If a fire is kept on the hearth in cold weather and in warm, a large lamp or candle is kept burning day and night, and the inner door always kept open, there is a continual draught passing along the floor towards the fire-place, which carries before it and up the chimney the most deleterious gases in the room, for the heaviest and the most poisonous of these are alwavs near the floor.

If the door cannot be left open all the time, it may be done for a few minutes at a time, every hour or two. In other cases, the window might be elevated. If this cannot be done without causing a draught of air upon the patient, provide a board two or three inches broad, and just as long as the window-sash; lay it on the sill lengthways, edge up, under the sash. This causes an opening at the joining of the upper and under sash, through which a draught of air is driven upward towards the ceiling, where it is warmed, and gives way downward to other cool air coming in constantly, thus giving an out-door air warmed to supply the patient all the time. If needed, additional bedclothing can be supplied, thus securing two most important conditions for the invalid: First, a pure out-door air to breathe. Second, comfortable bodily warmth.

6. There should be no standing liquid of any description in a sick room, not even the purest cold water; because, like a pane of glass of a very cold morning, the cold water causes the tainted atmosphere of the sick room to settle on its surface, and condense into oily drops, which under a microscope exhibit various impurities, even yellow matter, to drink which would be disgusting. If not drank, the same particles are made gaseous by the warm air of the room, are evaporated, mingled with the air, and breathed into the lungs, to be incorporated directly with the blood.

7. Everything perishable by evaporation should be removed from the room, as food and fruits of every description, because their smell, their exhalations, contaminate the air; every atom helps to make it less pure.

8. All medicines, bottles, and vials, or anything else which reminds of medicine, should be kept out of sight, except at the moment of administering them.

9. There should be no clothing hanging about in the room, and as little drapery about the bed and windows as possible, for these hold dust and absorb odors, and keep them in the room. For the same reason it is usually better to have no carpet on the floor, except a strip alongside of the bed, for carpets retain dust and dampness; and if any liquid is spilled on them, it is not dried for days, and odors accumulate. Still, if the patient is quite ill, or greatly debilitated, or has been ill for a long time, a carpet is indispensable, as it deadens the sound of feet and moving of chairs, which would otherwise interfere with the quiet and sleep of the patient. In all diseases sleep is better than medicine, and promotes cure more than anything else.

10. The qualifications of the nurse are so varied and so important, that great care should be taken, and no expense spared, to secure a good one. Women are generally better than men; although, if the patient is pretty helpless, and requires to be moved in and out of bed much, strength is a requisite.

The nurse should be either specially attached to the invalid, or should be a friendly acquaintance, with whom the patient has had pleasant associations, for these things secure a more devoted attention than hired services. At the same time, intelligent capability and experience are to be preferred in a stranger to pure affection only.

The nurse should be firm, and quietly decided; of a kindly, gentle nature, and sympathetic; not hasty or touchy, but calm and deliberate; at the same time spry, active, prompt. And not least, by any means, but of the very first importance, there should be a cheerful confidence and composure in the expression of the face all the time.

There are many things which the nurse should not do, — which she should studiously avoid. And to do this properly a good judgment is indispensable. She should not wear a silk dress, or any other fabric which gives a rustling noise; for repose and quiet are essential in sickness, and whatever disturbs a doze, or wakes from sleep, or rouses up, just as sleep is coming on, is a great misfortune.

All short, sharp, sudden movements, should be watched against, as they tend to alarm the patient. All suddenness should be avoided, all impatient or alarmed exclamations, everything which has the slightest tendency to discompose the invalid.

Mere visitors should not be allowed to remain in the sick room more than five minutes, just long enough to allow a friendly greeting, and the expression of a hope that soon all will be well again, with the communication of such intelligence as might make a pleasant impression on the mind. Avoid questioning very sick persons as to the state of the health. They seldom know exactly how they are; are rarely good judges of their condition. They may feel as if they did not know whether they were any better or worse, and the very effort to come to a truthful decision in their own minds, is at times quite exhausting, especially if the questions are repeated by the nurse or every visitor. A good nurse will know how the patient is without inquiry a great deal better than the patient, than convalescents especially; for as persons are getting well, particularly from serious or long illness, they are very desponding, and often think they are going to die, when it is clear to the physician and nurse that the crisis is just over, and that recovery is quite certain.

Nor should the nurse ask the patient if anything is wanted, or what he will have. Nine times out of ten the sick don't know what they want, and instinctively avoid the effort of deciding what they want. Besides, they are very apt to want what it would be very unwise to give. Then it is mere trifling to ask the question.

A competent nurse can anticipate a patient's needs, leaving wants out of the question; can tell better than the patient what is required, and what is best to be done.

Whatever is said to a patient should be as hopeful and assuring as is compatible with truthfulness; and it is only necessary to suggest, that every expression should be avoided which is calculated to excite apprehension or even disquietude, such as "You have changed a great deal," "I would not have known you," "You are very sick," "I am afraid you will not get well;" and yet these things have been said to the sick.

Never whisper in a sick room, or speak in a low tone. Let everything uttered be in a clear voice, distinctly enunciated, so that the patient may hear it. This self-evident propriety is too often disregarded by even rational people. Never look sideways at a patient, or seem to be scanning him. On the other hand, never stare. The moment you are out of conversation, leave the room. It is not uncommon to see a person sit with the eyes fixed on the patient for one, two, or more minutes, not knowing what to say, compelling the sick one to keep up the conversation; or, if they do talk, the remarks are of the most commonplace kind.

Never wake a patient, even to give medicine or to dress a wound, for sleeping is a healing process, giving strength and life. In almost all sickness, a tendency to sleep is a favorable sign.

Let the patient alone. This is said in reference to many who wait on the sick who are incessantly doing something, either fixing up the room, adjusting the bedclothes, or making some change, all of which tend to break up the quiet so necessary to an invalid. On the other hand, if the patient is in his right mind, and seems disposed to talk, or is inclined to ask questions, then it is advantageous to engage in conversation, but do not allow it to fall into a line of despondency. The good nurse will know how to lead the way to subjects which are interesting and cheering.

It is better to have two nurses in serious cases, so as to alternate several times in the twenty-four hours, for a sleepy or an overtaxed nurse can easily kill a patient whose life is hanging by a thread.

Avoid as much as possible bringing in a new nurse, especially a stranger. It discomposes the patient. The introduction into the house of a new servant is disquieting to healthy people.

It is better to have a woman to nurse a man. There is a gentleness, a sympathy, in woman. There is a magnetism in her touch, in the softness of her voice; a carrying away the mind to mother and sister, which is peculiarly grateful to a sick man. Besides, a woman has that instinctiveness of perception of what is needed, of what would add to the patient's comfort in a thousand little ways, which is of infinite advantage.

On the other hand, a man nurse is best for a woman, who feels all the time as if she wanted strength under her, firmness. It is best for a sick woman to feel all the time she "must." She will bend to a man's will; she would to a woman's, never. She imperatively needs confidence, reliance, some one to look to. This gives courage and hopefulness, very necessary elements as helps in recovery from disease.

The patient should have all the clothing of the body changed once a day; and if possible, the bed should be made night and morning. And several times during the day and night the bedclothes should be raised up from the body and let fall again, so as to drive out the confined air; or they should be thrown back and down towards the feet, to allow a full airing, while both bedclothes and body clothing should be kept most scrupulously clean. If at any time a drop of blood or other soiling should take place, the garment should be changed.

Prevent all surprises to a patient, especially disagreeable ones. To this end allow no one not of the household to enter a sick room without first acquainting the sufferer, and getting permission, ascertaining also that it is a hearty assent.

The nurse owes several duties to the medical attendant:—

First. Be able to give a clear, plain, and connected history of the case since his last visit. Mention all striking occurrences; if instructions have not been strictly followed; if the medicines have not been taken as directed, or if taken, rejected before they could operate; notice the color, quantity, and consistence of all the bodily discharges, and if possible, especially in critical cases, preserve them, outside the doors of the house, for inspection. There are times and conditions when life depends on these things.

Second. Notice whether the patient sleeps much or little, or if there is any tendency to being out of his mind, to mutterings, or moanings, or sighs.

Nursing is an art, a science, an acquisition. Many a time a disease is conquered, but the patient fails to get well for want of proper attention, from injudiciousness in eating; to avoid which a good nurse will always make it a point to ascertain clearly and distinctly from the physician what the patient is to eat, how often and how much, until he returns.

As a very general rule, sick persons should not eat oftener than at intervals of five hours, unless so weak that but very little, if anything, can be taken at a time. Then it may be better to eat oftener, at less intervals, but these are rare exceptions, and it is better to let the physician mark them out. One rule is always applicable: take but a few mouthfuls of any strange or unusual food or drink. Require the patient to eat slowly, and cut up all food very fine. Meats especially should be in pieces not larger than a pea, for then they are more easily dissolved in the juices of the stomach, as small bits of ice are sooner melted in a glass of water than large ones.

RECAPITULATION.

1. Be quiet.
2. Be decided.
3. Be composed.
4. Be firm.
5. Be sympathetic.
6. Consult your patient's wants, but do it as seldom as possible, to save him from the mental labor of comparing and weighing reasons, and deciding for himself.
7. Never seem to compel your patient to do anything, but lead him to do what is right; this is the triumph of good management.
8. Be clear in all your communications, and distinct in every word or syllable you say, without being loud or severe. The very effort at listening is sometimes exhaustive of the little strength the invalid has.

9. Never peep, nor poke, nor pother.
10. No trickeries nor deceptions in the sick chamber.
11. Be always on the alert.
12. Sameness is terrible to one who has been long an invalid; hence, make some change in the room every day; not many, one or two at a time. Either change the position of some one or two things already in the room, or introduce a picture or painting, or even a bunch of flowers; these often enliven a room very much; at one time on the mantel, at another on the table, or in the window. Flowers of bright colors are best, or placed in among green sprigs, avoiding those of a strong or coarse odor. If a picture, let it be lively; let it be of the springtime, of childhood, or pastures green, with the lambkins or the little chickens; something which will remind of youth and innocence. These may appear to be small matters to the well and hearty, but to the poor invalid, who has been at death's door for weary weeks and months, and is just rising up to light and life again, they have an incalculable value, and for their sake and good, especial attention should be given to the suggestions.

